# Comparative analysis of dietary fiber impact on bile acid metabolism and gut microbiota composition in mice

**DOI:** 10.1038/s44355-025-00041-z

**Published:** 2025-10-24

**Authors:** Andrea Zöchling, Joana Séneca, Petra Pjevac, Arturo Auñon-Lopez, Qendrim Zebeli, Marc Pignitter, Kalina Duszka

**Affiliations:** 1 Department of Nutritional Sciences, Faculty of Lifesciences, University of Vienna, Vienna, Austria; 2 Joint Microbiome Facility of the Medical University of Vienna and the University of Vienna, Vienna, Austria; 3 Centre for Microbiology and Environmental Systems Science, Department of Microbiology and Ecosystem Science, Division of Microbial Ecology, University of Vienna, Vienna, Austria; 4 Institute of Physiological Chemistry, Faculty of Chemistry, University of Vienna, Vienna, Austria; 5 Vienna Doctoral School in Chemistry (DoSChem), Faculty of Chemistry, University of Vienna, Vienna, Austria; 6 Centre for Animal Nutrition and Welfare, University of Veterinary Medicine, Vienna, Austria

**Keywords:** Microbiology, Gastroenterology

## Abstract

Dietary fiber is essential for health but remains under-consumed in Western diets. Fiber types differ in their physicochemical properties, which influence gastrointestinal function, bile acid (BA) metabolism, and gut microbiota composition. C57Bl/6 mice were fed control or 10% (w/w) fiber diets containing cellulose, chitin, resistant starch, pectin, inulin, β-glucan, psyllium, dextrin, or raffinose. All fibers reduced bacterial diversity, while most increased *Akkermansia muciniphila* abundance. Cellulose/chitin and inulin/β-glucan/raffinose formed distinct microbiome clusters. *Rikenellaceae* correlated positively with taurine-conjugated BAs levels. BA concentrations were reduced across tissues. Taurine conjugates showed inverse liver-intestine distribution. Inulin and β-glucan resulted in the highest taurine conjugate levels and reduced intestinal taurine-conjugated BAs concentrations, suggesting enhanced bile salt hydrolase (BSH) activity. Resistant starch had a minimal effect. Psyllium most strongly impacted BA- and taurine-related gene expression, cecum size and weight loss. Dietary fibers distinctly modulate BA metabolism and gut microbiota, with implications for metabolic health and targeted therapies.

## Introduction

The recommended daily intake of 20–30 grams of dietary fiber is rarely achieved by the population in Western countries^1^. As part of a balanced diet, fiber consumption modulates appetite, improves glycemic control and supports diabetes management, and contributes to diminishing the risk of cardiovascular diseases by reducing total blood cholesterol levels and hypertension^2–5^. Additionally, it aids in maintaining the gastrointestinal (GI) tract health by fueling beneficial gut bacteria growth, reducing the risk of diverticular disease, as well as esophageal, gastric, and colorectal cancer^1,6–8^. However, diet delivers a wide range of fibers that differ in water solubility, viscosity, and physiological parameters, such as fermentability. Depending on these properties, the impact of fiber on nutrient absorption, microbiota composition and activity, postbiotics production, physiological changes (e.g., pH lowering), peristaltic-transit time, chyme and fecal volume, as well as mechanical irritation of the mucosal layer will vary^9,10^.

One of the mechanisms by which fiber may beneficially affect the consumer is orchestrated by its effects on bile acids (BA). A commonly recognized property of fiber is the sequestration of BA, which prevents BA reabsorption, thus promoting its secretion with feces. By reducing the BA pool, fiber increases de novo BA synthesis from cholesterol^11^. The capacity for various types of fiber to bind BAs varies and corresponds to the degree of their hypocholesterolemic effect^1,11^. Furthermore, fiber intake shapes the composition of GI microbial communities, which play a vital role in BA deconjugation and subsequent metabolism^12^. As an energy source, fiber promotes the proliferation of specific bacterial strains. Moreover, serving as a substrate for microbial fermentation, fiber results in the production of various metabolites, further impacting the residing gut microbiome as well as the host^13^.

Our previous research has shown that in mice, caloric restriction stimulates hepatic BA synthesis, conjugation to taurine, and secretion^14^. In the GI tract, BAs undergo modification by bacterial bile salt hydrolase (BSH), leading to the release of deconjugated BAs and free taurine^15–17^. Notably, mice submitted to caloric restriction tend to consume cage bedding to ease hunger, and cage bedding-derived fiber stimulates microbial BSH activity^17–19^. Correspondingly, a high-fiber diet, particularly when supplemented with soluble fiber, enhances BA deconjugation and increases intestinal taurine conjugate levels^17,18^.

To follow up on these findings, the current study explores the impact of dietary fiber on gut microbiota composition, BAs, and taurine. The selected fibers represent a spectrum of physicochemical properties that influence their physiological effects: insoluble fibers (cellulose (low fermentability), chitin, resistant starch (partially fermentable starch)), soluble, viscous fibers (pectin (highly fermentable), psyllium (partially fermentable), β-glucan (moderately fermentable)), and soluble, non-viscous fibers (inulin (highly fermentable), dextrin (moderately fermentable), raffinose (rapidly fermentable)). These fiber types were selected to investigate how distinct physicochemical properties influence microbiota composition.

BA levels, composition, and taurine levels were analyzed in different compartments, aiming to understand the fiber’s properties regarding BA binding, stimulation of synthesis, secretion, deconjugation, and metabolism.

## Results

### Distinct fiber types differentially impact body and cecum weight

**Fig. 1:**
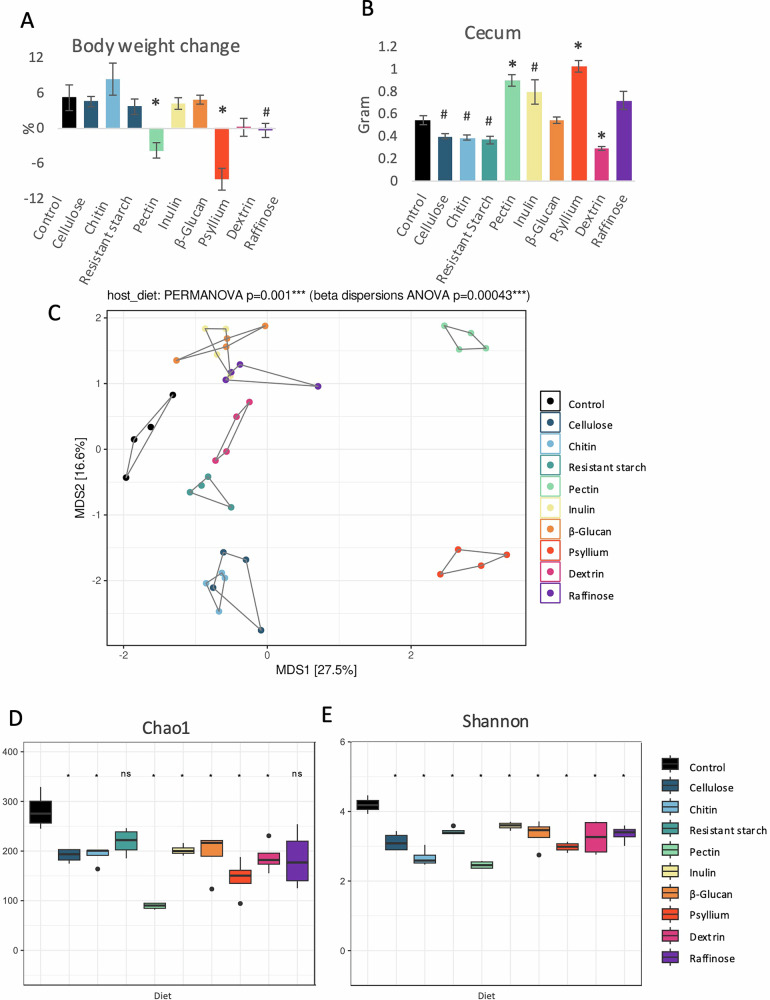
The impact of various types of fiber on mice body parameters and fecal bacteria composition. Body weight changes (**A**) and cecum weight (**B**) were measured in mice fed control or one of the high-fiber diets. Non-metric multidimensional scaling (MDS) (**C**), Chao1 index (**D**), and Shannon index (**E**) analyses were performed to identify the impact of the diets on fecal microbiota composition (Genus level). For panels A and B, an ANOVA was applied to assess statistical differences between the groups. For panels **C** and **D**, Wilcox tests were conducted between every diet and the control group, and p-values were corrected for the false discovery (FDR) rate. For panel **C**, PERMANOVAs were conducted between time points (shapes) and between diet types (colors) within each time point. * indicates statistical significance at *p* < 0.05; # indicates a strong trend with *p* < 0.05 higher than the threshold set at 0.0055, accounting for the correction for multiple testing; *n* = 4–5. Error bars represent ±SEM.

During the 14-day experiment, mice were fed a control diet or a high-fiber diet containing cellulose, chitin, resistant starch, pectin, inulin, β-glucan, psyllium, dextrin, or raffinose. Animals fed a diet rich in pectin or psyllium lost a significant amount of body weight, and raffinose introduced a trend toward reduced body weight compared to the control group (Fig. 1A). The body weight of mice fed other high-fiber diets did not statistically significantly differ from the control.

The weight of the cecum with its content was strongly affected by the diets (Fig. 1B). Groups consuming insoluble fiber (cellulose, chitin, and resistant starch diets) showed a trend towards a decreased cecum weight, and dextrin reduced the weight statistically significantly compared to the control group (Fig. 1B). Pectin and psyllium diets caused an increase in cecum weight compared to the control, and inulin resulted in a similar trend (Fig. 1B). The impact on cecum weight was even more substantial when adjusting for body weight, with pectin and psyllium resulting in approximately two times higher cecum weight (3.8% and 4.6%, respectively) as a percentage of total body weight (Supplementary Fig. S1A).

### Fiber-rich diets result in distinct fecal microbiota composition profiles

Before the intervention, no differences in fecal microbial community structure and diversity between mice assigned to different experimental groups were observed (Supplementary Fig. S1B–D). After the consumption of high-fiber diets, substantial diet-specific changes in bacterial richness, diversity, and community structure were detected (Fig. 1C, Supplementary Fig. S1C–D). All of the diets besides resistant starch and raffinose statistically significantly decreased microbiota richness compared to the control at the end of the treatment (Fig. 1D). Shannon diversity was significantly reduced by all diets compared to the control (Fig. 1E). The microbial community structure was the most distinct from the controls in pectin and psyllium-consuming animals (Fig. 1C). Inulin, β-glucan, and raffinose groups, as well as cellulose and chitin, created two clusters indicating the closest overlap in bacteria composition (Fig. 1C).

**Fig. 2:**
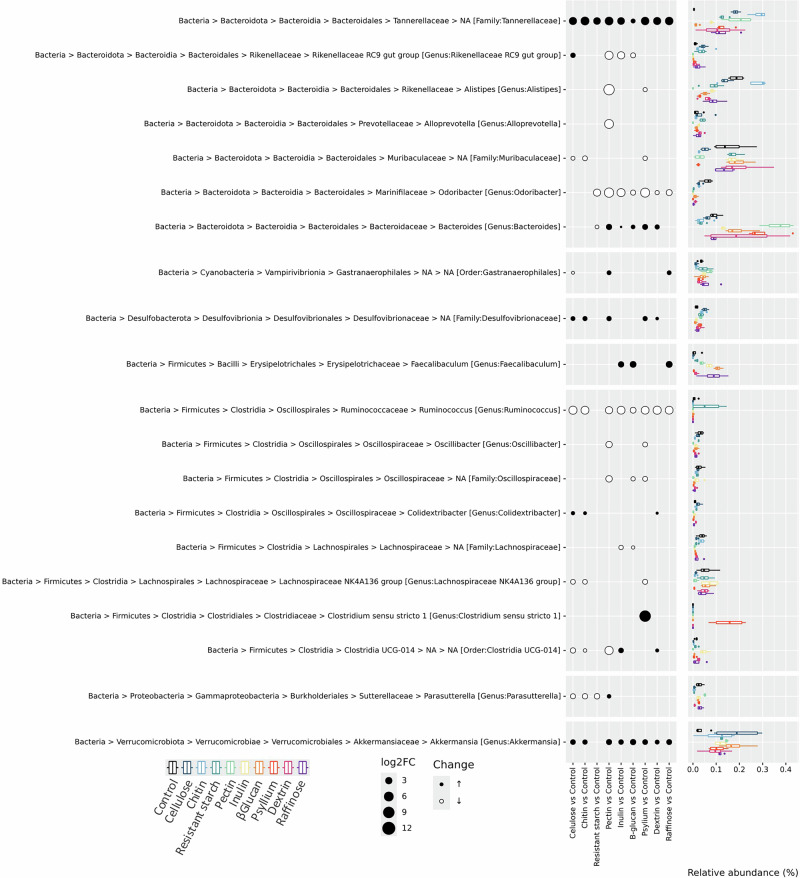
The impact of fiber on fecal microbiota. Differentially abundant bacterial genera between diet groups and the control group at the end of the experiment. The circles represent the log2fold change of bacterial genera between groups, and the color of the circle represents an increase or decrease compared to the control group. The barplots indicate the relative abundance of bacterial genera across all diet groups. Only bacterial genera that were statistically significantly differentially abundant across all pairwise combinations are shown.

All of the high-fiber diets introduced an increase in the relative abundance of members of the family *Tannarellaceae* (Fig. 2, Supplementary Fig. S1D). Additionally, all but resistant starch-consuming mice displayed significantly higher relative abundances of genus *Akkermansia* and reduced abundance levels of genus *Ruminococcus* compared to the control group (Fig. 2, Supplementary Fig. S1D). Also, the microbiota profiles for pectin and psyllium diets were similar, with the primary difference being a strong increase in *Clostridium Sensu stricto 1 ASV* for the psyllium diet (Fig. 2).

### Fiber mainly reduces BA concentration and affects its composition in the liver, intestinal mucosa, and feces

**Fig. 3:**
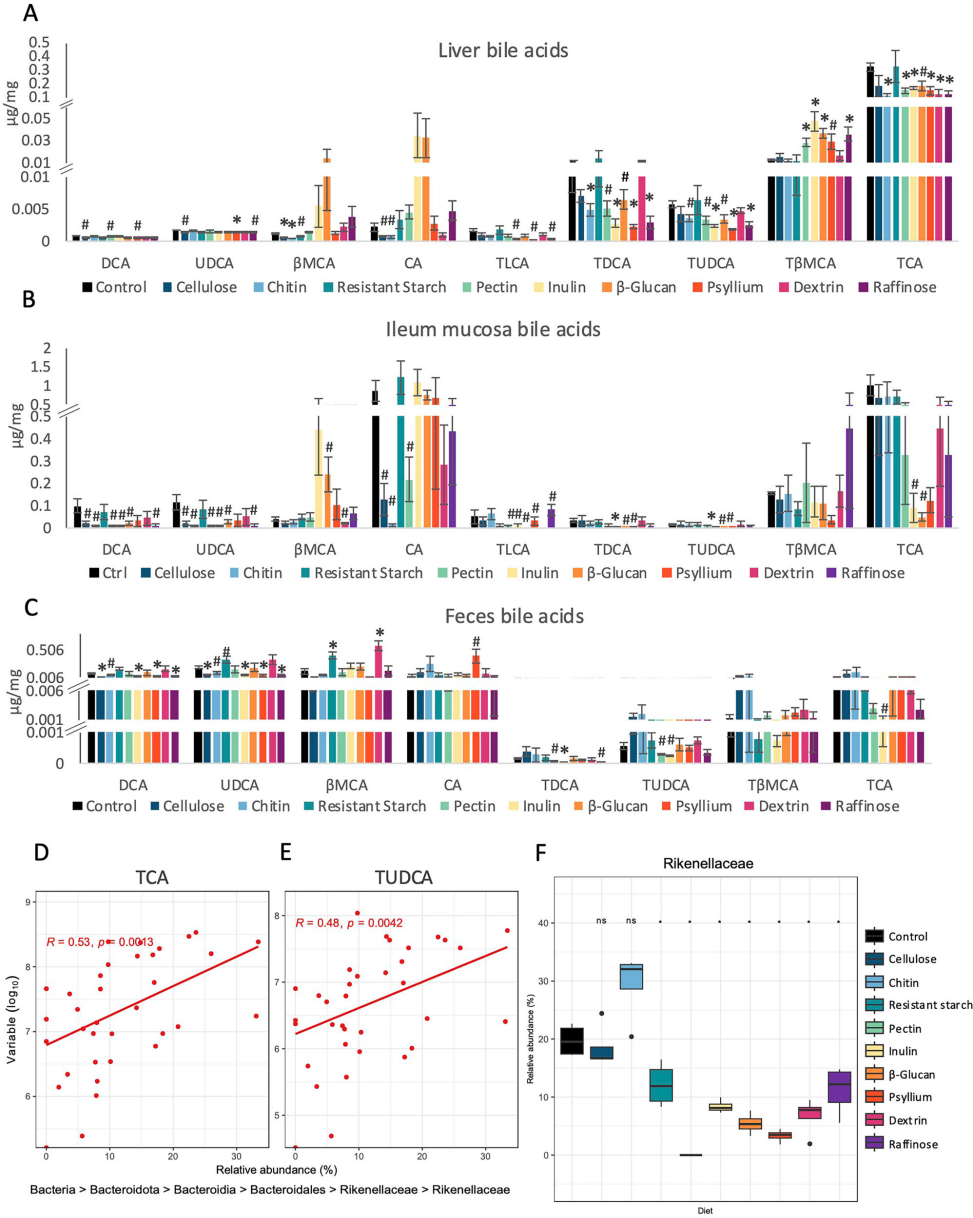
The impact of high-fiber diets on bile acids (BA) levels and composition. BA concentration was measured in the liver (**A**), mucosa of ileum (**B**) and feces (C) using HPLC-MS/MS. Bile acids: CA: cholic acid; DCA: deoxycholic acid; βMCA: β-muricholic acid TCA: taurocholic acid; TDCA: taurodeoxycholic acid; TLCA: taurolithocholic acid; TβMCA: tauro-β-muricholic acid; TUDCA: tauroursodeoxycholic acid; UDCA ursodeoxycholic acid. **D**, **E** Significant Spearman’s correlation coefficient (Rho) between the abundance of family *Rikenellaceae* and TCA and TUDCA. **F** Relative abundance of family *Rikenellaceae* in all diet groups after the end of the treatment. Asterisks denote statistically significant differences (*p* < 0.05) between each diet group and the control group. All p-values were FDR corrected. For **A**–**E**, ANOVA was used to verify statistical differences between the groups. * symbols statistical significance; # specifies a trend with *p* < 0.05 higher than the threshold set at 0.0055, accounting for the correction for multiple testing. For **F**, wilcox tests was used to compare the groups and * indicates a statistically significant result at *p* < 0.05 after the correction for multiple testing. Bars represent the mean of four to five biological replicates ±SEM.

In the liver, chitin, pectin, inulin, psyllium, and raffinose had the most consistent statistically significant or close to significant impact on the levels of BAs (Fig. 3A). Resistant starch and dextrin showed the smallest impact, with only one type of BA, β-muricholic acid (βMCA), and taurocholic acid (TCA), respectively, being affected statistically significantly or showing a statistical trend (Fig. 3A). In cases of nearly all affected BAs, fiber diets reduced the levels of BAs compared to the control diet. The only exception was tauro-β-muricholic acid (TβMCA), of which levels were increased (Fig. 3A).

In the mucosa of the ileum, fewer statistically significant changes were observed than in the liver (Fig. 3B). Significant changes or trends pointed towards a reduction in taurine-conjugated BAs compared to the control group, particularly for inulin, β-glucan, and psyllium diets. Regarding deconjugated BAs, the concentration of βMCA showed an increasing trend in the β-glucan group compared to the control group. All the other changes concerning deconjugated BAs implied reduced BA levels; however, none of them were statistically significant. Interestingly, inulin, β-glucan, and psyllium tended to have the same repressing impact for both BAs counterparts of the pairs deoxycholic acid (DCA) and taurodeoxycholic acid (TDCA), as well as ursodeoxycholic acid (UDCA) and tauroursodeoxycholic acid (TUDCA) (Fig. 3B).

In the feces, inulin, psyllium, and raffinose had the most consistent impact, lowering levels of several BAs (Fig. 3C). Meanwhile, β-glucan did not affect any BAs, and dextrin only increased the concentration of βMCA (Fig. 3C).

Overall, BA levels were primarily decreased across all types of samples without a distinctive fiber-specific pattern. The BA concentration was consistently the least affected by resistant starch across the three tested types of samples. Additionally, the comparison of the BA pool following their transit from the liver to the intestine and feces pictures a gradual decrease in the ratio of taurine-conjugated to deconjugated BAs (Fig. 3A–C).

Correlations between BA and relative abundances of ASVs clustered at the family level revealed a significant positive correlation between TCA and TUDCA and family *Rikenellaceae* (Fig. 3D, E). The relative abundance of *Rikenellaceae* was significantly reduced in samples from cellulose, resistant starch, pectin, inulin, β-glucan, psyllium, dextrin, and raffinose-consuming mice compared to the control group (Fig. 3F).

### Fiber-associated changes in taurine concentration are complementary in the liver and ileum mucosa

**Fig. 4:**
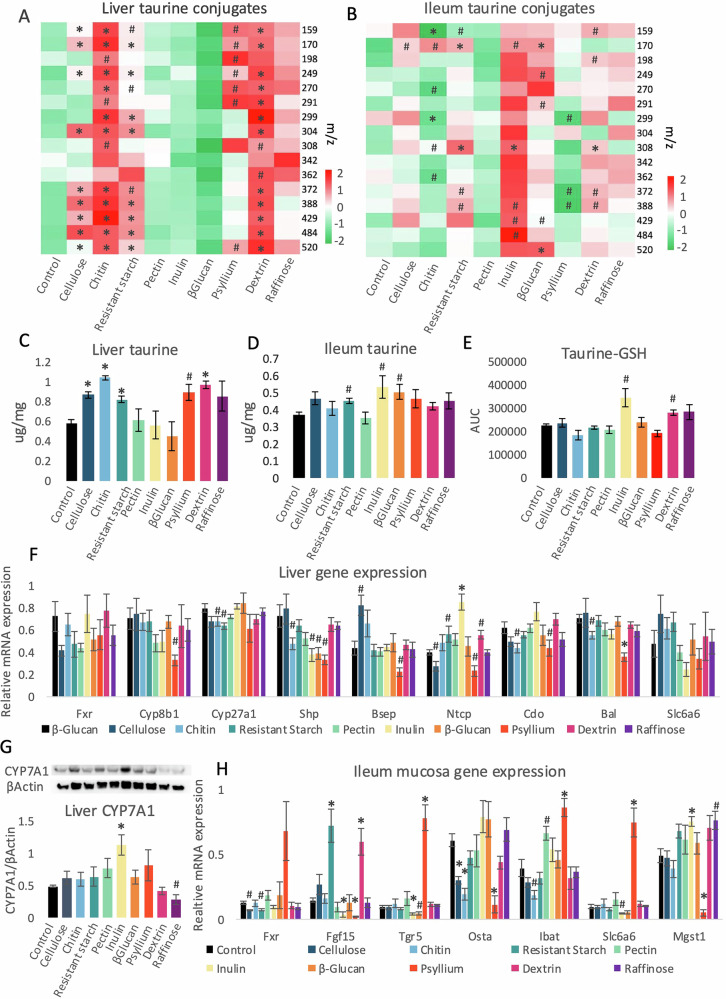
The impact of high-fiber diets on taurine and expression of BA- and taurine-related factors. The levels of taurine conjugates (**A**, **B**), free taurine (**C**, **D**), and taurine-glutathione (GSH) conjugate (**E**) were assessed in the liver (**A**, **C**) and ileum mucosa (**B**, **D**, and **E**) using HPLC-MS/MS. Gene expression was measured in the liver (**F**) and the ileum mucosa (**H**) with qRT-PCR. Genes: *Bal*: bile acid CoA ligase*; Bsep:* bile salt export pump; *Cdo*: cysteine dioxygenase; *Cyp8b1:* sterol 12-α-hydroxylase*; Cyp27a1:* sterol-27-hydroxylase*; Fgf15:* fibroblast growth factor 15; *Fxr*: farnesoid X receptor; *Ibat:* ileal bile acid transporter*; Mgst1:* Microsomal Glutathione S-Transferase 1; *Ntcp*: Na + /taurocholate cotransporting polypeptide; *Ostα:* organic solute transporter α*; Shp*: small heterodimer partner; *Slc6a6:* Solute Carrier Family 6 Member 6 (taurine transporter)*; Tgr5:* takeda G protein-coupled receptor 5. CYP7A1 (cholesterol 7-α-hydroxylase) and β-Actin protein levels were verified with western blot and quantified using ImageJ software (**G**). ANOVA was applied to assess statistical differences between the groups. * represents statistical significance, # indicates a trend (0.0055 < *p* < 0.05); *n* = 4–5. Error bars stand for ±SEM.

Since taurine plays a vital role in the conjugation of hepatic BAs and their deconjugation by gut microbiota, taurine and its conjugate levels were measured in the liver and intestinal mucosa. Changes in conjugated taurine levels (Fig. 4A, B) corresponded to the concentration of free taurine (Fig. 4C, D) in both the liver (Fig. 4A, C) and ileum mucosa (Fig. 4B, D). Compared to control, increased hepatic levels of taurine and some of its conjugates were measured for cellulose, chitin, resistant starch, psyllium, and dextrin diet groups (Fig. 4A, C). While in the ileum mucosa, resistant starch, inulin, and β-glucan diets tended to increase free taurine levels and showed corresponding impact on several taurine conjugates (Fig. 4B, D), with some implications for taurine-glutathione (GSH) conjugate (Fig. 4E). Overall, the liver and ileum showed complementary levels of taurine and/or its conjugates with higher hepatic levels in groups consuming insoluble fiber (cellulose, chitin, and resistant starch) as well as psyllium, dextrin, and raffinose diets. Correspondingly, chitin and psyllium tended to reduce ileal taurine conjugate levels. Inulin and β-glucan, which did not introduce a change in the liver, resulted in the strongest rise in the intestinal taurine conjugate levels. Accordingly, the trend in the increase in free and conjugated taurine levels (Fig. 4B, D) corresponds to the trend in the reduction of taurine-conjugated BAs (taurolithocholic acid (TLCA), TDCA, TUDCA, and TCA) in the intestine (Fig. 3B).

To help distinguish between the impact of the host and microbiota on BA and taurine levels, the expression of BA and taurine-related factors was assessed in the liver and intestinal mucosa (Fig. 4F–H). The expression of *Fxr* in the liver was not affected by any of the diets (Fig. 4F). Psyllium, as well as chitin and resistant starch diets, resulted in statistical trends toward the reduction of hepatic *Cyp27a1* and *Cyp8b1* gene expression, respectively (Fig. 4F). However, the protein levels of the primary BA synthesis regulator, CYP7A1, increased in the liver of mice consuming a diet high in inulin and reduced for the raffinose group (Fig. 4G). The mRNA expression of *Shp*, the inhibitory factor for BA synthesis, showed a trend towards reduced levels for chitin, inulin, β-glucan, and psyllium diet groups. Genes coding for BA transporters *Bsep* and *Ntcp* tended to be affected by several of the diets; however, only the stimulatory impact of inulin on *Ntcp* was statistically significant. Genes coding for taurine synthesis (*Cdo*) and conjugation to BAs (*Bal*) enzymes were similarly impacted by chitin and psyllium. Finally, the mRNA levels of the taurine transporter *Slc6a6* were not influenced by the diet (Fig. 4F). Overall, the impact of the diets on hepatic gene expression was mild, and psyllium affected more genes than other types of fiber.

The impact of the fiber diets on gene expression was stronger in the intestinal mucosa than in the liver (Fig. 4F, H). The most striking changes were observed in the intestinal mucosa for the psyllium group, which affected all of the tested genes. Inulin was the second most impactful diet and it affected the mRNA levels of *Fgf15*, *Tgr5*, and *Mgst1*, and resulted in a trend toward changes in *Slc6a6* expression. The expression of *Mgst1* (Fig. 4H) corresponded to the increase in taurine-GSH conjugate (Fig. 4E). Overall, psyllium tended to affect hepatic and intestinal gene expression most profoundly.

## Discussion

Dietary fiber influences body weight by several mechanisms. Soluble fiber serves as a substrate for SCFA production^7,20–22^, and an energy source for the host (2 kcal/100 g)^23^, while simultaneously reducing nutrient digestibility by limiting enzyme access and affecting digestive transit time^24^. The presented data showed that soluble fiber promoted weight loss in mice, with pectin and psyllium showing the most pronounced effects. These high-viscosity fibers form gel-like masses in the GI tract, encapsulating nutrients and preventing absorption while increasing cecum size. The observed weight loss may also result from increased energy requirements for peristaltic contractions needed to move fiber masses through the digestive tract, though quantitative data on this mechanism remain limited.

Previous research indicates that soluble fiber consumption increases BSH activity^17^. Bacterial strains with particularly high BSH activity reduce plasma cholesterol levels^25,26^ and limit the emulsification of dietary fats, thus restricting energy availability^27^. Moreover, deconjugated BA cholic acid (CA), enhances weight loss in mice through TGR5 signaling-mediated increase in brown fat tissue metabolic rate^28^. Accordingly, we observe increased expression of TGR5 in the intestine of psyllium diet-fed mice, which experienced the highest body weight loss. Finally, taurine deconjugated from BA is preferentially taken up by white adipose tissue, where it supports fat loss during CR^19^. These microbiota-mediated fiber effects may partially explain the observed body weight changes.

An enlarged cecum accompanies the consumption of a high-fiber diet^6,29,30^ and ingestion of cage bedding by energy-restricted mice^31^. Additionally, in germ-free mice, it indicates aggregation of fiber mass due to the lack of microbial degradation^32–34^. Our results show that cecum size depends on fiber type, with some fibers actually reducing cecum weight, suggesting differential microbial processing capabilities.

Fiber impacts BA composition by stimulating hepatic BA synthesis, microbial BA modification (deconjugation and metabolism), as well as sequestration and secretion of BAs with feces. Moreover, the intestinal content encloses BAs directly secreted from the gall bladder as well as microbiota-metabolized BAs^11–13^ and the capacity to reabsorb BAs depends on the type of BA and GI tract section^35^. Therefore, in the presented experiment, liver BAs were measured to picture synthesis and recirculation, ileum mucosa to verify BAs composition following the modification by gut microbiota and absorption, while fecal samples reflect sequestration and the composition of unabsorbed BAs.

Overall, gene expression analysis suggests a stronger impact of the fiber on the regulation of BA- and taurine-related genes in the intestine and relatively mild changes in the liver. Notably, psyllium influenced the expression of all of the tested genes in the intestine, and it also had the most substantial impact of all fibers on hepatic gene expression.

While fecal BA concentrations showed modest differences between groups, this result reflects concentration per gram wet weight rather than total excretion. The comparable intestinal reabsorption capacity across groups, as demonstrated by BA levels in the intestinal mucosa, suggests that fiber regulates BA removal primarily by increasing fecal mass rather than altering reabsorption efficiency^36^.

In a rat study by Ide T. et al.^37^, consumption of diets containing 10% of various water-soluble fibers (citrus pectin, konjak mannan, and guar gum) reduced hepatic concentration of taurine and CDO activity, while insoluble dietary fibers (cellulose, corn bran, and chitin) had a reverse impact. Our study confirms the impact on hepatic taurine levels for cellulose and chitin, while pectin did not show any impact. Importantly, Ide T. et al.^37^ used a diet containing a mix of several types of soluble fiber, and as we show, various soluble types of fiber have distinct impacts on taurine levels in the liver, which might explain the partial discrepancy in results.

Interestingly, there was an inverse pattern between hepatic and intestinal taurine conjugate levels, particularly for cellulose, chitin, resistant starch, psyllium, and dextrin, increasing hepatic taurine while decreasing intestinal levels, whereas inulin and β-glucan showed the opposite pattern. Additionally, hepatic *Cdo* and *Bal* gene expression - involved in taurine synthesis and conjugation, respectively - tended to be reduced in the groups that had abundant taurine in the liver, while low taurine levels did not trigger differences that would be statistically relevant. Hepatic taurine levels reflected the balance between synthesis, export, and conjugation to BA^38^. The lack of expression changes of hepatic taurine transporter suggests no impact on free taurine export. While the progressive shift from taurine-conjugated BAs in the liver to deconjugated forms in the ileum and feces demonstrates active microbial BSH activity. Fiber, particularly the soluble type, stimulates BSH activity^17,18,39^. In accordance with previous studies, inulin^39^ and β-glucan^18^ tended to increase free taurine and taurine conjugate levels in the intestinal mucosa. Equally, in our study, the increased levels of free taurine in the intestine associated with inulin and β-glucan consumption correspond with increased deconjugated BA and reduced taurine conjugates (TLCA, TDCA, TUDCA, and TCA), consistent with enhanced BSH activity.

BSH is an abundant enzyme found in all major phyla within the gut microbiota^40^, likely due to its benefits for bile resistance and GI colonization success^27,41^. For the host, probiotics with high BSH activity trigger metabolic profile changes, suggesting protection against atherosclerotic disease^42^. Thus, identifying factors affecting BSH activity may become valuable for therapeutic purposes. Importantly, various BA types have distinct impacts on microbiota composition, and taurine itself also affects it^43^. Thus, the correlation between conjugated and deconjugated BAs and microbiota reflects not only BSH activity but also the impact of free taurine and BA on gut bacteria. Therefore, to definitively conclude whether fiber triggers changes in BSH activity, an enzymatic assay is needed.

Following deconjugation from BAs in the intestine, taurine creates conjugates, among others, with GSH^15,44,45^. In calorie-restricted mice, taurine-GSH formation coincides with increased GSH-S-transferases (GST) expression and activity^15,45^. Similarly, in the current study, inulin consumption resulted in the highest taurine and taurine conjugate levels, corresponding with increased *Mgst1* (GST) expression and taurine-GSH conjugate level in the intestinal mucosa. Moreover, alike calorie-restricted mice, inulin did not impact hepatic taurine levels. β-glucan showed a similar but less pronounced phenotype. Further parallels between inulin and caloric restriction include increased CYP7A1 levels and the trend towards reduced *Shp* expression^14^. However, contrary to calorie-restricted mice, inulin did not affect Cyp8b1 and Cyp27a1 mRNA levels, and except for TβMCA, hepatic BA levels remained unchanged^14^.

All tested high-fiber diets, except for resistant starch, significantly increased *Akkermansia muciniphila* abundance. The abundance of this mucin-degrading and butyrate-producing bacterium inversely correlates with obesity and metabolic disorders^46^. As a probiotic target for type 2 diabetes treatment, *A. muciniphila* stimulates incretin hormone secretion (glucagon-like peptide 1 (GLP-1), glucagon-like peptide 2 (GLP-2), and peptide YY (PYY))^47^. Moreover, *A. muciniphila* improves intestinal wall integrity by increasing mucin production, protecting the intestinal epithelium from LPS, and stimulating the production of anti-inflammatory cytokines^47^. A recent report indicated an important aspect of the co-utilization of dietary fibers by colonic mucin-consuming bacteria. Additionally, the relative abundance of *Bacteroides*, of which several strains are recognized as mucin consumers (i.e., *Bacteroides thetaiotaomicron*, *Bacteroides fragilis*, *Bacteroides caccae*)^8,48^, also showed an increase for several fiber types in our study.

When analyzing sequencing data, microbiota composition clustered according to fiber solubility and fermentability, with cellulose/chitin forming one cluster and inulin/β-glucan/raffinose another. Psyllium, pectin, and dextrin, despite being soluble, created distinct profiles, likely due to differences in fermentability and viscosity. Regardless of similarities between some of the fibers, there is no consistent impact on any of the measured parameters, and each fiber must be studied individually for its specific outcomes. Further studies focusing on fiber-related microbial metabolome are needed to understand the mechanism behind here reported impact on gut bacteria.

The presented study highlights the role of different fibers in modulating BAs, taurine metabolism, and gut microbiota composition, with potential translational implications for disease therapies, particularly those related to obesity, metabolic syndrome, and gastrointestinal health. Given that soluble fibers, such as inulin and β-glucan, promote changes in BAs and increase the relative abundance of gut microbiome members linked to improved metabolic health^42,46,47,49–51^, future studies could explore their therapeutic potential in conditions like type 2 diabetes, dyslipidemia, and fatty liver disease. Another promising research line includes investigating taurine levels and BSH activity modulation by fibers, whereby “functional foods” target lipid metabolism, energy regulation, and microbiome-driven immune responses. Additionally, fiber-based therapies could be developed as a form of personalized nutrition approaches that match individual microbiota profiles with optimal fiber types to maximize benefits for gut health and metabolic disorders. Targeted fiber blends would be compiled based on individual microbiota composition and imbalances, BA and cholesterol levels, gut and metabolic health, or BSH activity.

Future studies should investigate long-term effects, fiber-specific formulations, and potential synergistic combinations of different fiber types. Mechanistic studies examining BSH activity modulation, taurine transporter regulation, and microbiome-immune system interactions will enhance our understanding of fiber-mediated health benefits.

## Materials and methods

### Animal care and experimental procedures

Male C57Bl/6 mice were purchased from Janvier Labs (Le Genest-Saint-Isle, France) and kept under a 12 h light/12 h dark cycle in standard specific-pathogen-free (SPF) conditions. The mice were housed in group cages with wooden bedding (Lignocel select; J. Rettenmaier & Söhne GmbH + Co KG; Vienna, Austria). At ten weeks of age, each mouse cage was randomly assigned to an experimental group and, from now on, fed a control diet or a diet containing 10% (w/w) of one of the following fibers: cellulose, chitin, resistant starch, pectin, inulin, β-glucan, psyllium, dextrin, or raffinose. Each diet was assigned five mice. The diets were custom-made by SSNIFF-Spezialdiäten GmbH (Soest, Germany), and their composition is presented in the Supplementary Table 1. The experimental groups did not significantly differ in body weight at the beginning of the experimental procedures. Sample size was determined using power analysis based on our previous studies showing that *n* = 5 mice per group provides 80% power to detect a 25% difference in BA concentrations with α = 0.05, considering the expected effect size and variability observed in previous animal experiments involving feeding high-fiber diets^17,19^.

The mice had free access to water throughout the experiments. Fecal samples from each mouse were collected before the diet intervention and within three days prior to the dissection. One mouse from the raffinose-diet group died of unexplained reasons during the experiment. Following 14 days of the diet intervention, all mice were euthanized by isoflurane overdose, with blood drawn by cardiac puncture. Food was removed 2 h before the dissection. The body and cecum weights were recorded. Intestinal mucosa and liver samples from each animal were snap-frozen and stored at −80 °C.

Animal experimentation protocols were approved by the Austrian Bundesministerium für Wissenschaft, Forschung und Wirtschaft, Referat für Tierversuche und Gentechnik (ID 2022-0.257.032). The experiments were carried out according to the Animal Welfare Act guidelines.

### LCMS detection of bile acid (BA), taurine, and taurine conjugates

Samples were processed and analyzed according to the protocol reported previously^14,15^. To each sample, nine times the volume of ice-cold 100% methanol along with 1.4 mm ceramic beads were added just prior to homogenization in the Precellys 24 Tissue Homogenizer (Bertin Instruments, Montigny-le-Bretonneux, France), operating at 5000 rpm for two 15-s intervals. Following this, samples were incubated on ice for 10 min while shaking, then vortexed and centrifuged at 18,000 g for 10 min at 4 °C. The supernatants were carefully transferred to Eppendorf tubes and subjected to an additional centrifugation for 10 min at 18,000 g at the same temperature. The final supernatants were then placed into HPLC vials for analysis with an LCMS-8040 Liquid Chromatograph Mass Spectrometer (Shimadzu Corporation, Kyoto, Japan), using an Atlantis T3 3 μm column (2.1 × 150 mm, Waters, Milford, MA, USA).

For taurine level assessment, samples were evaluated in negative mode. Solvents A and B consisted of water with 0.1% formic acid and acetonitrile with 0.1% formic acid, respectively. The gradient was set at 40 °C, starting at 5% B for 2.5 min, increasing to 20% B at 8 min, and returning to 5% B at 9 min, followed by a one-minute hold. To estimate BA concentration, samples were analyzed in positive mode. Solvent A was water, while solvent B was a mixture of acetonitrile and methanol (3/1, v/v), both containing 0.1% formic acid and 20 mmol/l ammonium acetate. The solvent gradient at 30 °C started at 30% B for 5 min, then increased to 100% B at 25 min and maintained for 20 min.

### Fecal microbiota characterization

Fecal pellets were collected before the dietary intervention and within three days prior to dissection from all experimental groups. Fresh pellets were quickly frozen and stored at −80 °C for future processing. DNA was extracted from the fecal samples using the QIAamp Fast DNA Stool Mini Kit (Qiagen, Germany), and the V4 hypervariable region of the bacterial and archaeal 16S rRNA gene was amplified using primers 515 F/806 R^52^. DNA extraction, sequencing, and the processing of raw data took place at the Joint Microbiome Facility of the Medical University of Vienna and the University of Vienna (project ID JMF-2303-06). Gene amplicon libraries were prepared according to previously outlined methods^53^ and sequenced on an Illumina MiSeq (2 × 300 bp). Amplicon pools were derived from the raw sequencing data through the FASTQ workflow in BaseSpace (Illumina) using default settings, and the raw data processing followed the approach outlined previously^53^. Demultiplexing was perfomed with the Python package demultiplex (Laros JFJ, github.com/jfjlaros/demultiplex), which permits one mismatch for barcodes and two mismatches for linkers and primers. Amplicon sequence variants (ASVs) were inferred using the DADA2 R package v1.42^54^, applying the recommended workflow^55^. FASTQ reads 1 and 2 were trimmed at 220 nt and 150 nt with allowed expected errors of 2. ASV sequences were then classified using DADA2 and the SILVA database SSU Ref NR 99 release 138.1 (10.5281/zenodo.4587955)^56^. Applying a confidence threshold of 0.5, we excluded ASVs that were either unclassified or identified as eukaryotes, mitochondria, or chloroplasts prior to downstream analysis. After this filtering step, only samples with a minimum of 3000 reads were kept for further examination.

Downstream analyses were performed using R v4.3.2 and Bioconductor v3.16 packages SummarizedExperiment v1.32, SingleCellExperiment v1.24, TreeSummarizedExperiment v2.8^57^, mia v1.8 (https://github.com/microbiome/mia), vegan v2.6-4 (https://CRAN.R-project.org/package=vegan), phyloseq v1.44^58^, microbiome v1.22 (http://microbiome.github.io), microViz v0.10.8^59^, and ALDEx2 v1.32^60^. Alpha diversity, including richness and diversity indices, was calculated on rarified data (3210 read pairs/sample) using the R packages vegan and mia. Beta diversity was evaluated through a PCoA performed with Aitchison distance, using the R package microViz. The differences among per-group centroids were analyzed via a PERMANOVA based on Aitchison distances, employing the R packages vegan and microViz. Pairwise differential abundance testing between conditions was conducted at the genus level using DESeq2, setting alpha at 0.05 and employing other default parameters, after incorporating a pseudocount of 1 to the data. Correlations between centered log-ratio transformed counts of the most prevalent bacterial families and clinical variables were assessed using ALDEx2’s correlation test with Spearman’s coefficient and FDR multiple testing correction.

### qPCR

RNA extraction from the liver and intestinal mucosa was performed with the peqGOLD HP RNA kit (peQlab, Erlangen, Germany). For reverse transcription the qScript cDNA Synthesis Kit (QunataBio, Beverly, MA, United States) was used. Quantitative real-time PCR (qRT-PCR) reaction was carried out using the QuantStudioTM 6 Flex Real-Time PCR System (Applied Biosystems, Life Technologies, Carlsbad, CA, USA) along with the Neogreen qPCR Master Mix (Neo Biotech, Nanterre, France). The Supplementary Table S2 lists the primer sequences.

### Western blot

To lyse 10 µg of liver tissue, RIPA buffer (10x, EMD Millipore, Billerica, MA, USA) with Pierce Phosphatase Inhibitor (Mini Tablets, Thermo Scientific, Rockford, IL, USA) and the protease inhibitor cocktail (cOmplete Mini, EDTA-free, Roche, Mannheim, Germany) was used. For the electrophoresis, the Precision Plus Protein™ standard (Bio-Rad, Carlsbad, CA, USA) was utilized, followed by electro-blotting through the Trans-Blot® Turbo Transfer System (Bio-Rad) utilizing PVDF membranes (Bio-Rad). After drying for one hour and blocking, the membranes were incubated with a primary anti-β-Actin antibody (#4970; Cell Signaling Technology, Danvers, MA, USA) or CYP7A1 (#TA375303, OriGene, Rockville, MD, USA) at a dilution of 1:1000 in 2.5% BSA TBST for 2 h at room temperature. The secondary anti-rabbit antibody (#7074; Cell Signaling Technology) was used at a 1:3000 dilution. The bands were visualized utilizing the SuperSignal West Dura solution (Thermo Scientific) and ChemiDoc XRS+ (Bio-Rad) and quantified with ImageJ (version 1.53). The relative expression of CYP7A1 was normalized to β-Actin as a reference standard. Data from four replicates for each experimental group were employed to calculate the mean result.

### Statistics

Heatmaps were created to compare taurine conjugate levels across experimental groups using Z-scored data, showing each group’s deviation from the mean. They were displayed with the MATLAB extension COVAIN, designed to cluster groups based on the similarity of metabolite occurrence patterns. For statistical analysis, except for sequencing data (Section 2.3), one-way ANOVA with Bonferroni correction for multiple tests was applied using Excel Analysis ToolPak. Each experimental group was compared to the control group.

## Supplementary information

Supplementary Information

## Data Availability

The 16S rRNA gene amplicon sequencing data have been deposited at the Sequence Read Archive under the BioProject accession PRJNA1226173.
