# Supplementary material for: Comparative analysis of dietary fiber impact on bile acid metabolism and gut microbiota composition in mice
Source: NPJ Gut Liver. 2025 Oct 24;2(1):26. doi: 10.1038/s44355-025-00041-z (PMC12552122; doi:10.1038/s44355-025-00041-z)
Supplement: Supplementary file 1 — Supplementary Information [file 44355_2025_41_MOESM1_ESM.pdf]

Supplementary Data

Supplementary Figures

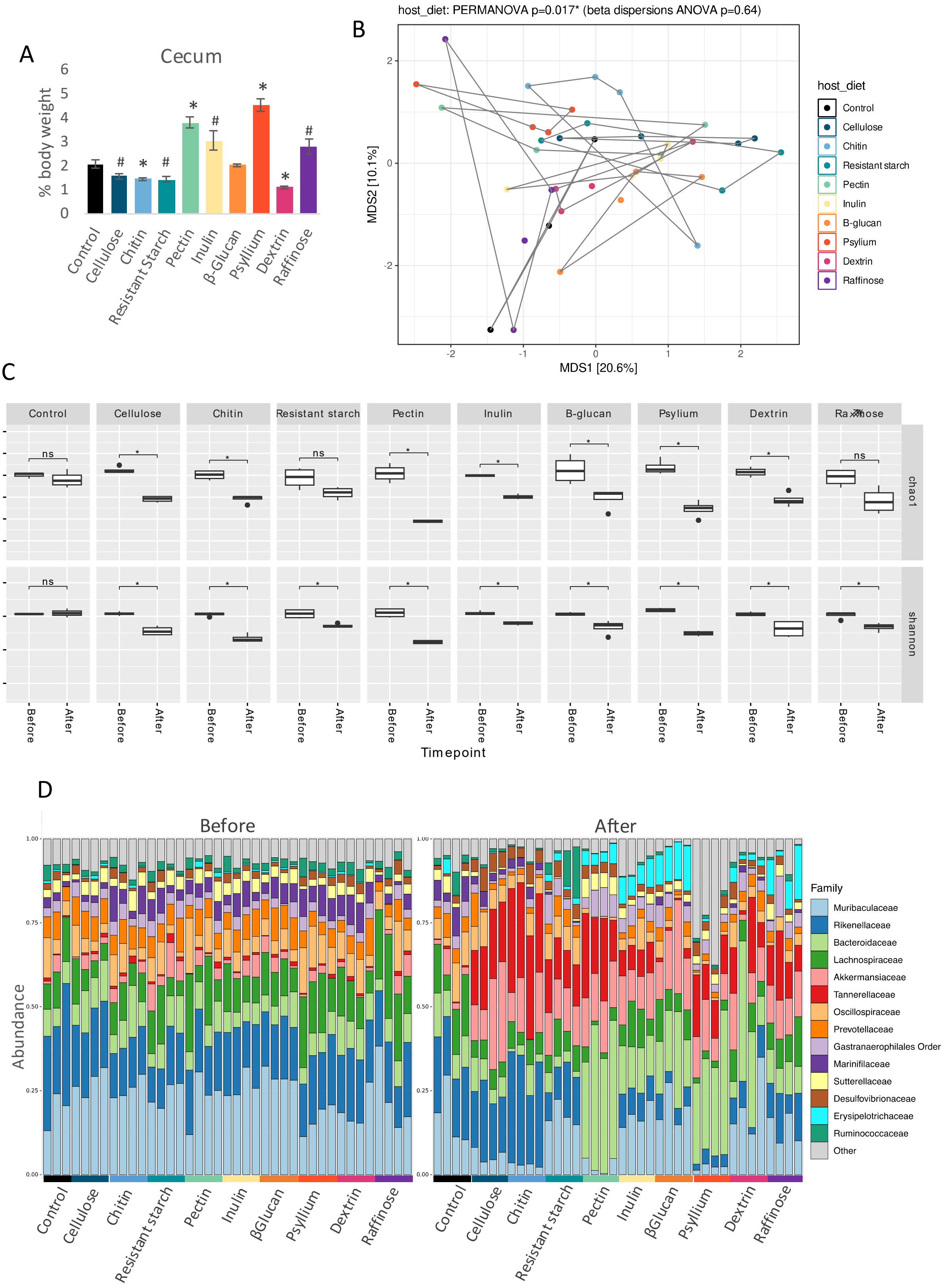

Supplementary Figure S1. Cecum weight was measured in mice fed control or one of high-fiber diets and presented as % body weight. * indicates statistical significance at $p<0.05$; # indicates a strong trend with $p<0.05$ higher than the threshold set at 0.0055, accounting for the correction for multiple testing (A). Non-metric multidimensional scaling (MDS) analysis was performed to compare the experimental groups before the diet treatment (B). Richness (Chao1) and diversity (Shannon Index) were analyzed between each group before and after the diet intervention. Significant differences ($p<0.05$) were assessed by a Wilcox test between all pairwise combinations. All p-values were FDR-corrected (C). Relative abundance of the top most abundant bacterial families before and after the diet intervention (D).

Supplementary Tables

Supplementary Table S1. Diet composition

	**Control**	**Fibre diets**
**Casein**	21.55	21.55
**Corn starch**	35	30
**Maltodextrin**	16	16
**Sucrose**	10	10
**Fibre**	5	10
**L-cysteine**	0.25	0.25
**Vitamin mix**	1	1
**Mineral mix**	6	6
**choline chloride**	0.2	0.2
**Soybean oil**	5	5

Supplementary Table S2. Sequence of primers used for qRT-PCR.

Gene ID	Forward	Reverse
**Eef1a1**	CCTGGCAAGCCCATGTGT	TCATGTCACGAACAGCAAAGC
**Bal**	TGTGTGTGAAGGAACCTGGA	ACCCGGACAACTTTGTGAAG
**Cdo**	GGGGACGAAGTCAACGTGG	ACCCCAGCACAGAATCATCAG
**Cyp8b1**	AGGCCAGTACTTCACCTTTG	CCTAACCAACAGCTTATGCC
**Cyp27a1**	AGACGATTGCCATCAAGGAC	GGGTTATCAGCCTCTTTCTTCCTCA
**Fgf15**	GGGGACGAAGTCAACGTGG	CTGGTCCTGGAGCTGTTCTC
**Fxr**	TCCGGACATTCAACCATCAC	TCACTGCACATCCCAGATCTC
**Ibat**	ACCACTTGCTCCACACTGCTT	CGTTCCTGAGTCAACCCACAT
**Ntcp**	AATCCAAGCTGCAGACGCACC	GCATCTTCTGTTGCAGCAGCCTT
**Mgst1**	CCTTCTCCCTGGATTCAGTCAT	TCGGCCATGCTTCCAATCTT
**Ostα**	CTTGACCTTTCTTACCACTGGCTCA	ACCAAAGCAGCAGAACACAGATACC
**Shp**	CTCATGGCCTCTACCCTCAA	GGTCACCTCAGCAAAAGCAT
**Slc6a6**	GCACACGGCCTGAAGATGA	ATTTTTGTAGCAGAGGTACGGG
**Tgr5**	GAGCGTCGCCCACCACTAGG	CGCTGATCACCCAGCCCCATG
